# HTLV-1-associated myelopathy/tropical spastic paraparesis (HAM/TSP) versus adult T-cell leukemia/lymphoma (ATLL)

**DOI:** 10.1186/s13104-021-05521-y

**Published:** 2021-03-23

**Authors:** Mohadeseh Zarei-Ghobadi, Mohsen Sheikhi, Majid Teymoori-Rad, Sahar Yaslianifard, Mehdi Norouzi, Somayeh Yaslianifard, Reza Faraji, Mohammad Farahmand, Shiva Bayat, Mohieddin Jafari, Sayed-Hamidreza Mozhgani

**Affiliations:** 1 Institute of Biochemistry and Biophysics, University of Tehran, Tehran, Iran; 2 Department of Biochemistry, Faculty of Life Sciences of Islamic, Azad University, Tehran north branch, Tehran, Iran; 3 Research Center for Clinical Virology, Tehran University of Medical Sciences, Tehran, Iran; 4 Department of Microbiology, School of Medicine, Alborz University of Medical Sciences, Karaj, Iran; 5 Dietary Supplements and Probiotic Research Center, Alborz University of Medical Sciences, Karaj, Iran; 6 Department of Medical Genetics, School of Medicine, Tehran University of Medical Sciences, Tehran, Iran; 7 Research Program in Systems Oncology, Faculty of Medicine, University of Helsinki, Helsinki, Finland; 8 Non-Communicable Diseases Research Center, Alborz University of Medical Sciences, Karaj, Iran

**Keywords:** Human T-lymphotropic virus type 1, HTLV-1 associated myelopathy/tropical spastic paraparesis, Adult T-cell leukemia/lymphoma, Pathogenesis, Systems virology

## Abstract

**Objectives:**

Human T cell leukemia virus-1 (HTLV-1) infection may lead to one or both diseases including HTLV-1-associated myelopathy/tropical spastic paraparesis (HAM/TSP) or adult T cell leukemia lymphoma (ATLL). The complete interactions of the virus with host cells in both diseases is yet to be determined. This study aims to construct an interaction network for distinct signaling pathways in these diseases based on finding differentially expressed genes (DEGs) between HAM/TSP and ATLL.

**Results:**

We identified 57 hub genes with higher criteria scores in the primary protein–protein interaction network (PPIN). The ontology-based enrichment analysis revealed following important terms: positive regulation of transcription from RNA polymerase II promoter, positive regulation of transcription from RNA polymerase II promoter involved in meiotic cell cycle and positive regulation of transcription from RNA polymerase II promoter by histone modification. The upregulated genes TNF, PIK3R1, HGF, NFKBIA, CTNNB1, ESR1, SMAD2, PPARG and downregulated genes VEGFA, TLR2, STAT3, TLR4, TP53, CHUK, SERPINE1, CREB1 and BRCA1 were commonly observed in all the three enriched terms in HAM/TSP vs. ATLL. The constructed interaction network was then visualized inside a mirrored map of signaling pathways for ATLL and HAM/TSP, so that the functions of hub genes were specified in both diseases.

**Supplementary Information:**

The online version contains supplementary material available at 10.1186/s13104-021-05521-y.

## Introduction

Infection with Human T cell leukemia virus-1 (HTLV-1) which normally takes an innocuous and insidious course in humans can rarely manifest as adult T cell leukemia/lymphoma (ATLL) and/or HTLV-1-associated myelopathy/tropical spastic paraparesis (HAM/TSP). HAM/TSP results in lower back pain, limb spasticity, progressive neurological decline, and urinary disturbances [1]. Contrastingly, ATLL is characterized by unrestrained growth of T-cell precursors in blood, bone marrow, thymus, or lymph nodes. Despite thorough investigations, it is still unclear what triggers HTLV-1 infection to remain innocuous or to progress to either of these complications [2, 3]. A thorough examination of the pathogenic pathways and the disruptions in host proteins and their interactions might therefore reveal novel aspects of the disease through identification of unbalanced pathways in HTLV-1 related diseases. Identifying the key dysregulated pathways could be utilized to screen individuals with higher risk and reveal novel therapeutic approaches to prevent the progression to either of these diseases. Analyzing the cellular gene expression signature in both of these complications highlights common and specific deregulated pathways in HTLV-1 related diseases which are likely to provide novel insight into the pathogenesis and the management of HTLV-1 related diseases.

Several analytical approaches and softwares have been developed (https://pubs.acs.org/doi/abs/10.1021/acs.jproteome.5b01080, https://academic.oup.com/bioinformatics/article-abstract/35/8/1436/5102873, https://journals.plos.org/plosone/article?id=10.1371/journal.pone.0189922) [4] as means to further analyze the high-throughput data derived from previous genomic studies to extract and conclude additional results which would only be available when the data from different studies were compared with each other. A well-known method to analyze previous datasets is to search for differentially expressed genes (DEGs) between normal individuals and those harboring the disease or between several complications associated with a single pathogen [5, 6].

In this study we performed the protein–protein interaction network-based analysis to determine DEGs between HAM/TSP and ATLL samples. The resulting networks and related hub genes were enriched in gene ontology for biological processes (BP) and the results and their implication is discussed.

## Main text

### Methodology

#### Microarray dataset

The gene expression profile GSE19080 was acquired from the public repository database gene expression omnibus (GEO) (www.ncbi.nlm.nih.gov/geo) which includes an individual platform, GPL9686. The dataset contains the results of microarray experiments using the human ImmuneArray cDNA array. The data related to ATLL (7 specimens) and HAM/TSP (12 specimens) patients were extracted and then analyzed.

#### Exploration of differentially expressed genes

The Data was first normalized and preprocessed with log2 transformation. Then, the differentially expressed genes (DEGs) and their value of fold changes (FC) were acquired by GEO2R, which is an interactive web tool based on GEOquery and limma packages in R computing language. The adjusted P-value < 0.05 (calculated by FDR) was selected as the criterion for selection of DEGs. The direction of dysregulation of each for DEGs was reported as upregulation (positive logFC) and downregulation (negative logFC) compared to baseline.

#### Construction of protein–protein interaction (PPI) network

In order to construct the PPI network, the online STRING (Search Tool for the Retrieval of Interacting Genes) database version 10.5 was employed [7]. The information and interactions from various biological sources, including physical interactions, functional association, high-throughput experiments, genomic context, co-expression, databases, and text-mining were considered. The cut-off criterion was set at combined score > 0.4 to analyze the PPINs.

#### Identification of hub genes

The network was analyzed by Network Analyzer app in Cytoscape (3.5.1) to calculate “degree” and “betweenness” centrality measures. The number of edges of a node is assigned as the degree [8] and the number of node visiting during moving all shortest paths is defined as betweenness centrality [9]. The genes with higher degree and betweenness scores were selected as hub genes. These genes and their associated PPIN were visualized using Gephi version 0.9.1 [10].

#### Gene ontology analysis

Gene ontology BP was assessed by Enrichr website [11]. Top ten major functional terms were selected based on z-scores for further analysis.

#### Signaling network analysis

The HTLV-1-implicated signaling network was depicted based on the KEGG and WikiPathway databases. The upregulated and downregulated genes were presented with red and blue coloring in visualized pathways, respectively.

### Results

#### Identification of DEGs and hub genes

The number of 1116 DEGs was recognized according to FDR < 0.05 by Benjamini–Hochberg procedure. Following the construction of the primary network by STRING, the network was analyzed by means of degree and betweenness centrality measures. 57 genes (Table 1) were selected as hub genes based on the aforementioned criteria. The logFC for each hub gene is reported in Additional file 1: Figure S1.

**Table 1:** List of the up-regulated (positive logFC) and down-regulated (negative logFC) hub genes in TSP vs. ATLL

TSP vs. ATLL			
Gene	logFC	Gene	logFC
TNF	2.08	CD44	− 0.3
ITGA2	1.97	VEGFA	− 0.38
PIK3R1	1.51	TLR2	− 0.41
CALM1	1.41	ACACB	− 0.42
PTK2	1.27	STAT3	− 0.46
HGF	1.25	TLR4	− 0.47
NFKBIA	1.22	EHMT2	− 0.61
PIK3CD	1.11	LYN	− 0.64
PRKCB	1.06	TP53	− 0.65
EZH2	0.99	CHUK	− 0.69
CTNNB1	0.9	KIT	− 0.71
GMPS	0.88	SERPINE1	− 0.75
IL8	0.81	CREB1	− 0.76
CAD	0.8	MAP3K1	− 0.76
YWHAZ	0.77	BTK	− 0.79
PPP2CA	0.76	TOP2A	− 0.8
NOS2	0.74	CD19	− 0.83
ESR1	0.74	SOCS3	− 0.85
SMAD2	0.72	SRC	− 0.86
RB1	0.68	BRCA1	− 0.91
NGF	0.66	IRS1	− 0.93
MAPK14	0.63	RANBP2	− 0.98
PTGS2	0.62	EEF2	− 0.98
PPARG	0.6	EGR1	− 0.99
CBL	0.58	TBP	− 1.25
PARP1	0.51	IGF1R	− 1.49
XPO1	0.48	APP	− 1.7
PHLPP2	0.47		
CDKN2A	0.4		
FN1	0.4		

#### PPIN construction

The relationship between hub genes was specified using STRING database. The network was constructed, as shown in Additional file 2: Figure S2. The network consists of 57 nodes and 716 edges. The size of each node was determined based on its degree value and the color of each node was specified according to their direction of deregulation with higher values of logFC represented by colors closer to red and lower values by blue.

#### Gene ontology biological processes

**Fig. 1:**
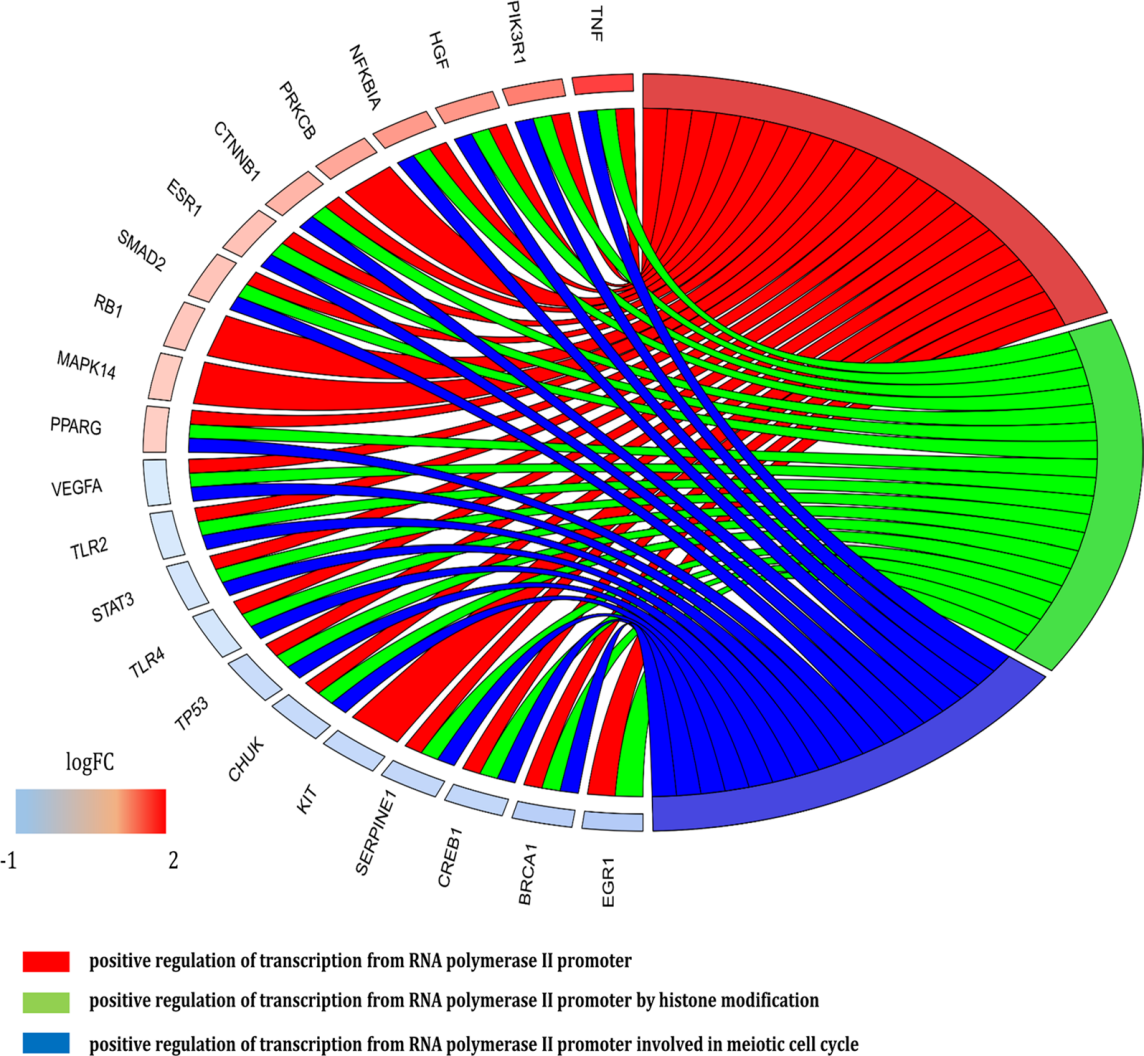
Circos plot of the common GO biological process between up-regulated and down-regulated genes in TSP vs. ATLL. The upregulated genes including TNF, PIK3R1, HGF, NFKBIA, CTNNB1, ESR1, SMAD2, PPARG and downregulated genes containing VEGFA, TLR2, STAT3, TLR4, TP53, CHUK, SERPINE1, CREB1 and BRCA1 were common in all the three enriched terms

The upregulated and downregulated hub genes were individually enriched in gene ontology BP and the top functional terms were selected. Additional file 3: Figure S3a, b reveals the BP of hub genes associated with upregulated and downregulated genes, respectively. The comparison between the Gene Ontology BP of the enriched upregulated and downregulated genes revealed that positive regulation of transcription from RNA polymerase II promoter (GO: 0045944), positive regulation of transcription from RNA polymerase II promoter involved in meiotic cell cycle (GO: 0010673) and positive regulation of transcription from RNA polymerase II promoter by histone modification (GO: 1903757) are common in the samples of this analysis. Figure 1 is a circos plot demonstrating the contribution amount of proteins in each of the GO BPs (GO: 0045944, GO: 0010673 and GO: 1903757). The upregulated genes including TNF, PIK3R1, HGF, NFKBIA, CTNNB1, ESR1, SMAD2, PPARG and downregulated genes comprised of VEGFA, TLR2, STAT3, TLR4, TP53, CHUK, SERPINE1, CREB1 and BRCA1 were commonly observed in all of the three enriched terms in TSP vs. ATLL.

#### Signaling network displaying differentiation between HAM/TSP and ATLL

**Fig. 2:**
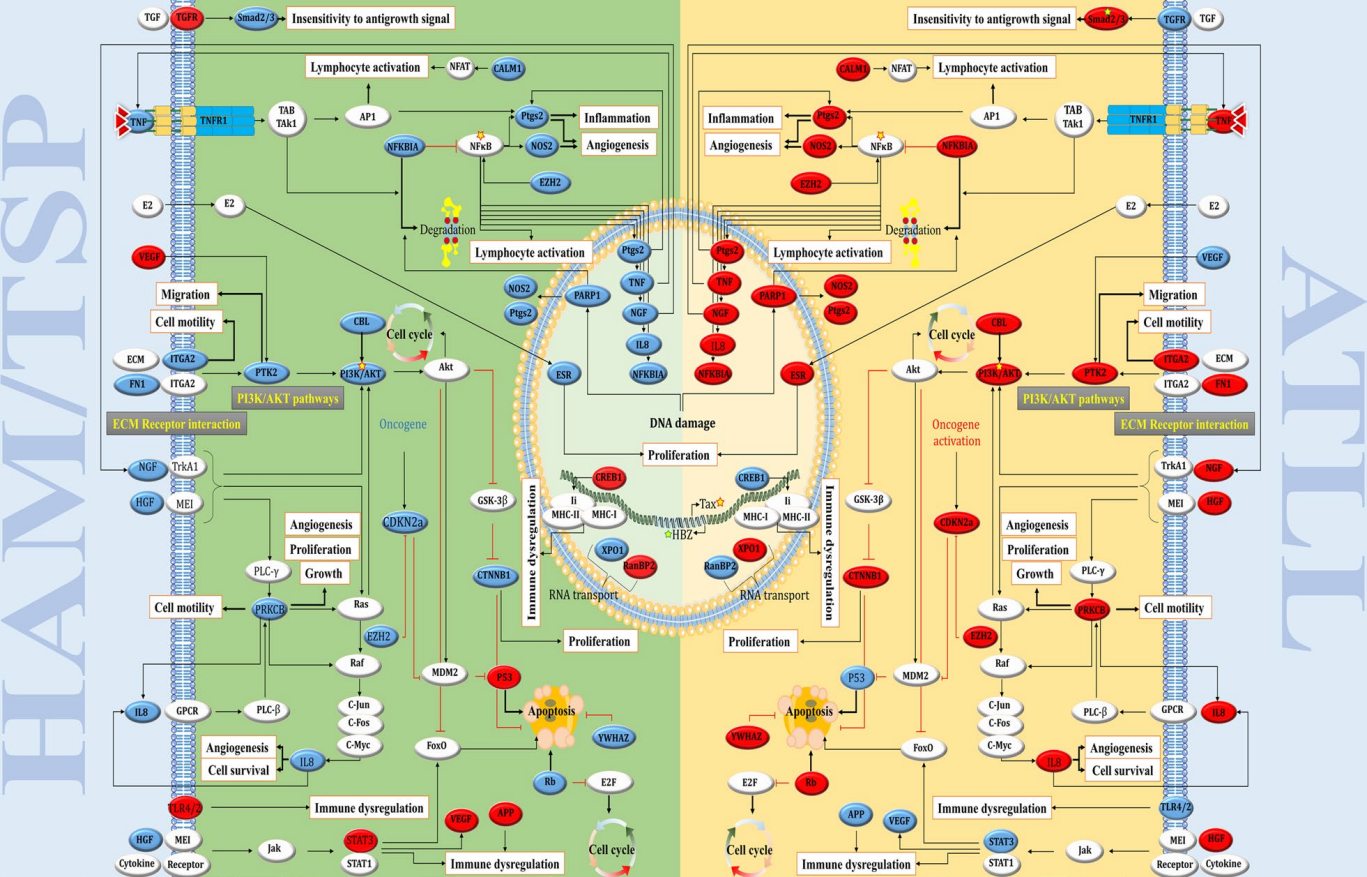
The proposed mirror-like signaling network for the pathogenesis of ATLL and HAM/TSP diseases. The upregulated and downregulated genes are identified by colors of red and blue, respectively. The signaling pathways were manually drawn according to the KEGG, WikiPathway, and literature reports

The related pathways and the connections between them are depicted in Fig. 2. The signaling network mapping for ATLL and HAM/TSP is illustrated mirror wise to provide a visual comparison of hub genes were specified in both diseases. The expression level of the following genes is increased in ATLL; TNF, AP1, NFAT, CLAM1, PI3K-AKT, PRKCB, NGF which is accompanied by spontaneous promotion of NF-κB pathway and persistent lymphocyte activation. In addition, the upregulation of PRKCB, ITGA2, IL8, and NOS2 genes is associated with inflammation, angiogenesis, cell survival, and migration pathways. Contrarily, promotion of pathways related to apoptosis and immune dysregulation are noticeable in HAM/TSP. The upregulation of genes including TP53, EGR1, Serpine1, IGFR1 can induce apoptosis, while the increase in STAT3, TLR2/4, MAP3K1, CREB1, and APP lead to disruption of the immune response.

### Discussion

The results of this analysis indicate high dissimalirities in regulation of cell proliferation and inflammatory pathways in ATLL and HAM/TSP. While the enriched pathways regarding RNA polymerase II [12] and DNA replication in this study just allude to a surge in clonal expansion or increased viral transcription of TATA-box containing 5′LTR of HTLV-1 [13] in the provirus-harboring cells in ATLL, the identified hub genes provide further insight into the pathogenesis of both diseases.

The SRC gene is highly overexpressed in a variety of human cancers [14]. In this study, however, ATLL was associated with lower levels of SRC expression. Counterintuitively, the loss of src homology 2 containing tyrosine phosphatase (SHP-1) is associated with spontaneous activation in HTLV-1 infected T-cells [15]. Furthermore, another src-like tyrosine kinase LYN, which is normally upregulated via Tax in HTLV-1 cell lines [16], is downregulated in this study compared to HAM/TSP. This suggests that the increased LYN expression is not maintained with the loss of Tax expression in later stages of ATLL development [17]. Further studies are needed to determine the role of src-like tyrosine kinases in ATLL and HAM/TSP.

One of the major pathways in ATLL is lymphocyte activation, which is of particular importance in ATLL compared to ACs and HAM/TSP [6]. Transduction of lymphocyte activation signals occurs through the following ways: (i) activation of NF-kappa B, (ii) increase the AP1 gene expression via up-regulation of TNF [18], and (iii) enhancement the expression of NFAT gene due to up-regulation of CALM1 [19]. The diminished expression of the aforementioned genes in HAM/TSP indicates their lesser significance compared to ATLL. Therefore, it could be speculated that NF-κB pathway is likely the major signaling pathways in ATLL compared to other forms of HTLV-1 infection, as inhibition of NF-κB by a super-repressor form of IκBα (SR-IκBα) in infected T-cells in ATLL results in cell death regardless of Tax expression [20]. Furthermore, there was a significant disruption of genes related to phosphatidylinositol 3-kinase-Protein kinase B (PI3K-AKT) pathway in ATLL (PIK3CD, PIK3R1, and IRS1) which has been demonstrated to have a role in activation of NF-κB pathway [21].

Several genes like FN1, ITGA, PTK2, HGF, and CBL can affect PI3K-AKT pathway in the focal adhesion and ECM Receptor interaction pathways [22–24]. Moreover, PI3K-AKT itself is indirectly activated through the ROS pathway. This pathway can be stimulated through upregulation of PRKCB due to functions of NGF and HGF which are in turn mediated by PLC-γ and PLC-β, respectively [25–27]. The increase in NF-κB action is also important in upregulation of PTGS2, TNF, NGF, IL8, and NFKBIA in ATLL [25, 28–31], as well as NOS2 [32, 33]. The NFKBIA increase in ATLL cells however, supposedly inhibit NF-κB pathway [28]. However, pathways such as PARP-1 that lead to NFKBIA degradation, are activated in ATLL to overcome the inhibition. The NF-κB pathway is less prominent in HAM/TSP, as the effective genes mentioned above are inversely expressed in it.

Inhibition of apoptosis is another pivotal distinction in signaling pathways between ATLL and HAM/TSP, marked by significant dowregulation of TP53 in ATLL [6]. TP53 loss or mutation in ATLL cells reflects their aggressive proliferation and poor prognosis as described in many other cancers [34, 35]. The upregulation of SMAD2 in ATLL facilitates metastasis and is associated with TP53 mutation [36].

Furthermore, MDM2 can increase the expression of CDKN2A, which in turn increases TP53 [37]. Although, upregulation of CDKN2A secondary to the induction of oncogenes is unavoidable, EZH2 functionally inhibits the role of CDKN2A in cell cycle [38]. The decrease in expression level of TP53 is accompanied by downregulation of its target genes including Serpin1 in ATLL. Serpin1 has been linked to reduction of tumor invasion and growth [39]. In addition, the downregulation of EGR1 tumor repressor is also observed in ATLL in this study. The increase in the expression level of EGR1 in HAM/TSP along with the higher levels of TP53 and Serpin1 compared to ATLL restricts the uncontrolled proliferation of infected cells as seen in ATLL [40]. Furthermore, the analysis revealed PRKCB, ITGA2, IL8, and NOS2 genes to be significantly upregulated in ATLL. The afforementioned genes are associated with cell motility and angiogenesis and could be surmised to contribute to the metastasic properties of leukemic cells in ATLL.

Viral persistence cannot continue without significant dysregulation of the host immune response. The balance of immune response can tip to either immunosuppression or hypersensitivity in HTLV-1 infections, resulting in ATLL and HAM/TSP, respectively. The mild immunodeficiency almost exclusively associated with ATLL is partly the consequence of disruption of regulatory genes implicated in the immune response. In this study, the expression levels of STAT3, TLR2/4, MAP3K1, CREB1, and APP genes in ATLL have been reportedly decreased, leading to reduced antigen presentation, T-cell costimulation, and immune response against the virus [41–45]. The different patterns of gene expression described throughout the article further elaborates the unique immunophenotype observed in each of these two diseases.

### Conclusion

This study provides a novel approach to gene expression analyses regarding HTLV-1 related diseases by comparing the gene expression signature between samples from HAM/TSP and ATLL patients. The results revealed distinct patterns of gene expression, especially in cell cycle regulation and immune response between the two diseases.

## Limitations

Further detailed studies help us understand other functions of the involved genes in the pathogenesis of HTLV-1.

## Supplementary Information

**Additional file 1: Figure S1.** List of the up-regulated (positive logFC) and down-regulated (negative logFC) hub genes in TSP vs. ATLL and vice versa.

**Additional file 2: Figure S2.** The PPINs formed between the identified hub genes of ATLL vs. TSP group. The node size is representative of the degree of nodes and the node color is indicative of the expression level of nodes ranging from red (upregulated genes) to blue (downregulated genes).

**Additional file 3: Figure S3.** The most meaningful GO biological process terms in top ranks of z-score were specified for two series of upregulated and downregulated genes.

## Data Availability

The dataset analyzed in this study [GSE19080] was accessed through gene expression omnibus public repository (www.ncbi.nlm.nih.gov/geo).

## Abbreviations

HTLV-1: Human T-lymphotropic virus type 1
HAM/TSP: HTLV-1-associated myelopathy/tropical spastic paraparesis
ATLL: Adult T-cell leukemia/lymphoma
GEO: Gene Expression Omnibus
DEGs: Differentially expressed genes
STRING: Search Tool for the Retrieval of Interacting Genes
PPIN: Protein–protein interaction network
KEGG: Kyoto Encyclopedia of Genes and Genomes
PI3K-AKT: Phosphatidylinositol 3-kinase-Protein kinase B
