# Supplementary figures and images for: HTLV-1-associated myelopathy/tropical spastic paraparesis (HAM/TSP) versus adult T-cell leukemia/lymphoma (ATLL)
Source: BMC Res Notes. 2021 Mar 23;14:109. doi: 10.1186/s13104-021-05521-y (PMC7989087; doi:10.1186/s13104-021-05521-y)

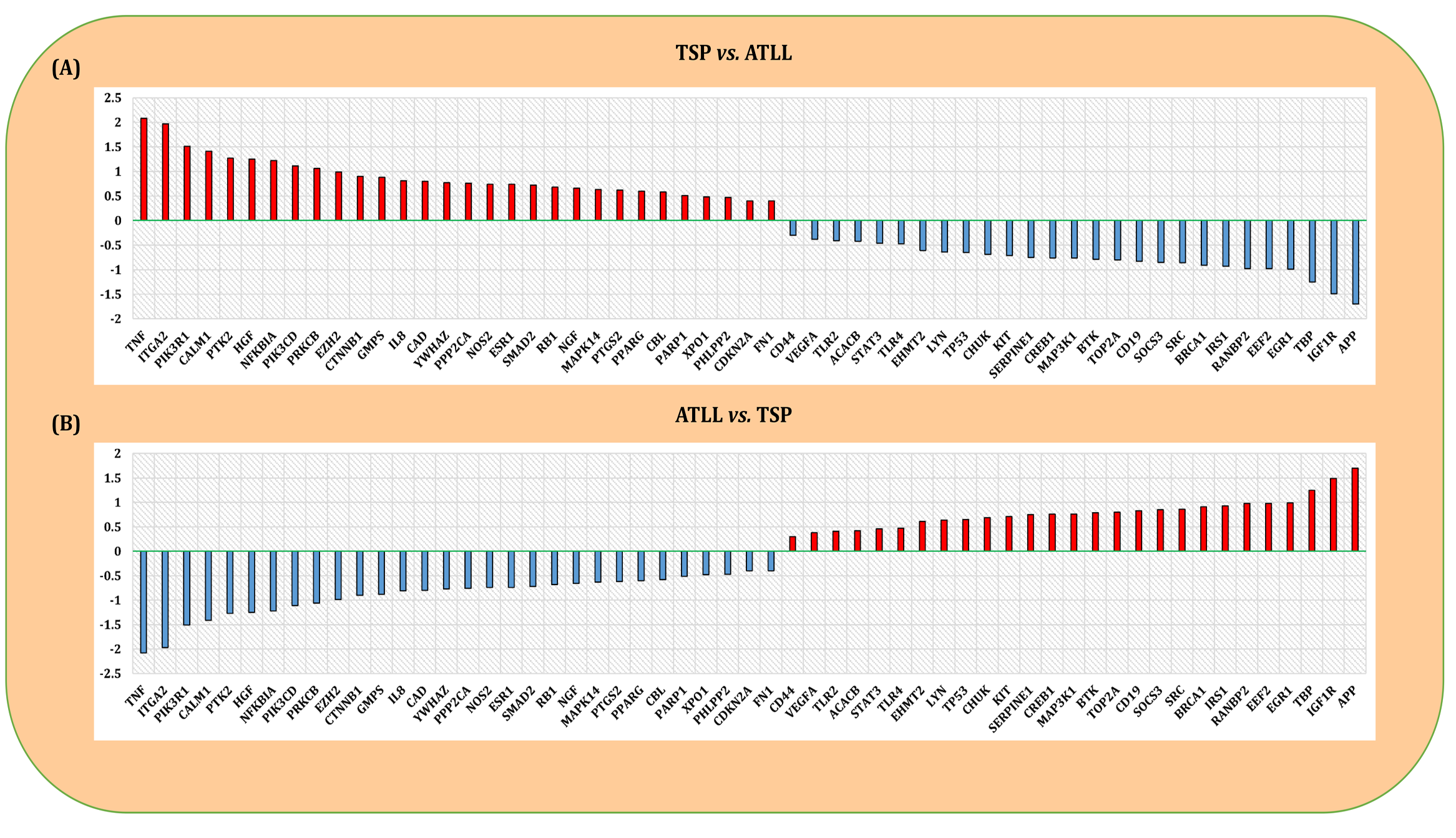

Supplement: Supplementary file 1 — Additional file 1: Figure S1. List of the up-regulated (positive logFC) and down-regulated (negative logFC) hub genes in TSP vs. ATLL and vice versa. [file 13104_2021_5521_MOESM1_ESM.tif]

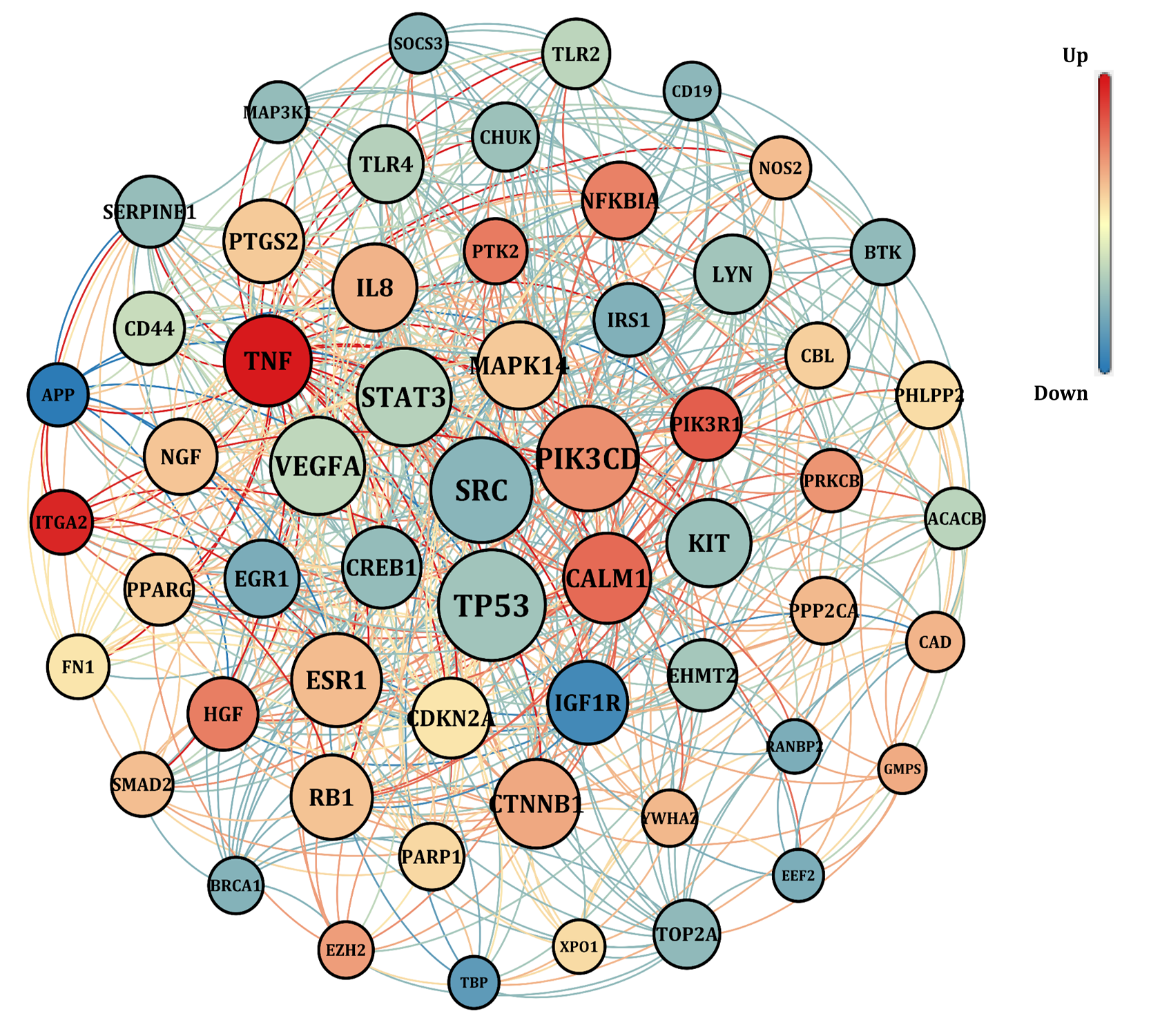

Supplement: Supplementary file 2 — Additional file 2: Figure S2. The PPINs formed between the identified hub genes of ATLL vs. TSP group. The node size is representative of the degree of nodes and the node color is indicative of the expression level of nodes ranging from red (upregulated genes) to blue (downregulated genes). [file 13104_2021_5521_MOESM2_ESM.tif]

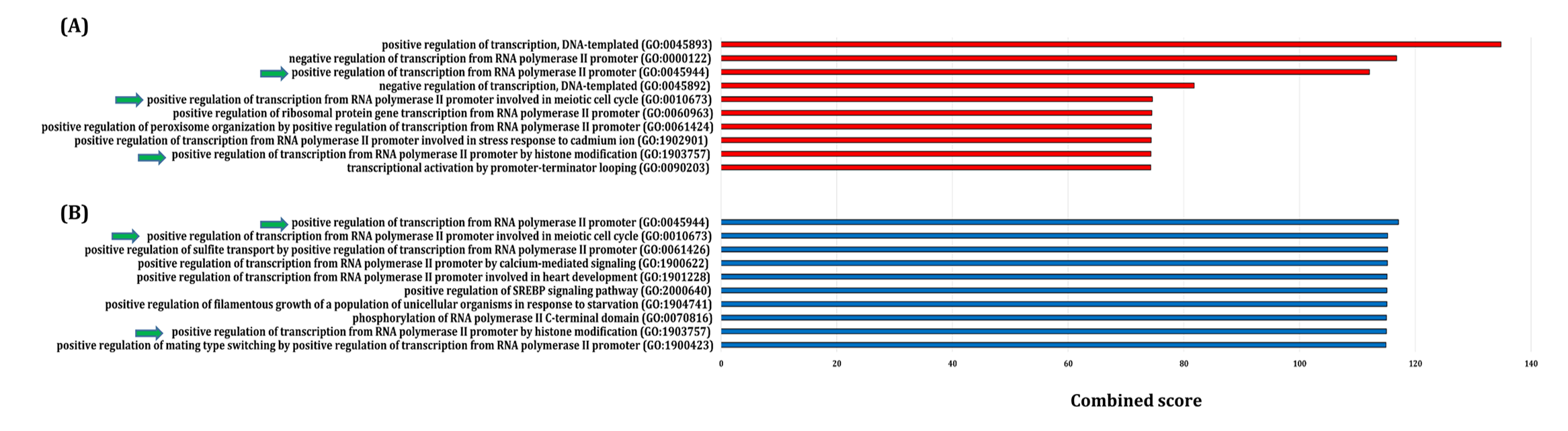

Supplement: Supplementary file 3 — Additional file 3: Figure S3. The most meaningful GO biological process terms in top ranks of z-score were specified for two series of upregulated and downregulated genes. [file 13104_2021_5521_MOESM3_ESM.tif]
